# Efficacy of Probiotics in Children and Adolescents With Atopic Dermatitis: A Randomized, Double-Blind, Placebo-Controlled Study

**DOI:** 10.3389/fnut.2021.833666

**Published:** 2022-01-26

**Authors:** Paula Danielle Santa Maria Albuquerque de Andrade, Jorgete Maria e Silva, Vanessa Carregaro, Laís Amorim Sacramento, Luciana Rodrigues Roberti, Davi Casale Aragon, Fabio Carmona, Pérsio Roxo-Junior

**Affiliations:** ^1^Department of Pediatrics, Ribeirão Preto Medical School, University of São Paulo, São Paulo, Brazil; ^2^Department of Immunology, Ribeirão Preto Medical School, University of São Paulo Ribeirão Preto, São Paulo, Brazil

**Keywords:** atopic dermatitis (AD), microbiota, immune tolerance, SCORing Atopic Dermatitis (SCORAD), probiotics

## Abstract

**Objective:**

To evaluate the clinical efficacy of a mixture of probiotics (Lactobacillus and Bifidobacterium) in children and adolescents with atopic dermatitis (AD) and the effects on sensitization, inflammation, and immunological tolerance.

**Methods:**

In this double-blind, randomized, placebo-controlled clinical trial, we enrolled 60 patients aged between 6 months and 19 years with mild, moderate, or severe AD, according to the criteria proposed by Hanifin and Rajka. Patients were stratified to receive one gram per day of probiotics or placebo for 6 months. The primary outcome was a decrease in SCORing Atopic Dermatitis (SCORAD). Secondary outcomes were to assess the role of probiotics on the use of topical and oral medicines (standard treatment), serum IgE levels, skin prick test (SPT), and tolerogenic and inflammatory cytokines. Background therapy was maintained.

**Results:**

Forty patients completed the study (24 probiotics, 16 placebo). After treatment for six months, the clinical response was significantly better in the probiotics group; the SCORAD decreased [mean difference (MD) 27.69 percentage points; 95% confidence interval (CI), 2.44–52.94], even after adjustment for co-variables (MD 32.33 percentage points; 95%CI, 5.52–59.13), especially from the third month of treatment on. The reduction of the SCORAD in probiotic group persisted for three more months after the treatment had been discontinued, even after adjustment for co-variables (MD 14.24 percentage points; 95%CI, 0.78–27.70). Patients in the probiotics group required topical immunosuppressant less frequently at 6 and 9 months. No significant changes were found for IgE levels, SPT and cytokines.

**Conclusions:**

Children and adolescents with AD presented a significant clinical response after 6 months with a mixture of probiotics (Lactobacillus rhamnosus, Lactobacillus acidophilus, Lactobacillus paracasei, and Bifidobacterium lactis. However, this clinical benefit is related to treatment duration. Probiotics should be considered as an adjuvant treatment for AD.

## Introduction

Atopic dermatitis (AD) is a chronic inflammatory, pruritic and relapsing skin disease that is commonly associated with other atopic comorbidities (1). The diagnostic of AD is based on clinical symptoms, whereas the SCORing for Atopic Dermatitis index (SCORAD) helps physicians to assess disease severity on a regular basis (2, 3).

Several studies have demonstrated that immunological tolerance is closely related to the composition of intestinal microbiota (4). Accumulating evidence has shown an association between microbial dysbiosis and the development of allergy during childhood (5).

The development and maturation of gut microbiota constitute a dynamic and non-random process, in which positive and negative interactions between key microbials take place. This process is influenced by many perinatal conditions, such as the mode of delivery, the type of feeding, and antibiotic use (6).

The microbiological profile of the gastrointestinal tract of children exclusively fed with breast milk is different from children fed with formula or in a mixed manner. Lactobacilli counts are higher in breastfed infants than formula-fed infants. The microbiological profile of the digestive tract of newborns who use formula can promote the development of allergic reactions, autoimmune diseases, and many other disease entities (7).

Furthermore, breastfeeding appears to moderate the detrimental effects of C-section delivery and intrapartum antibiotics on the early microbiota, producing a microbiota profile more similar to that of vaginally-delivered infants or those not receiving antibiotics (8). Exclusive breastfeeding is essential in this case (7).

Probiotics are live microorganisms whose immunomodulatory effects and clinical benefits are promising in the treatment of various diseases, given adequate administration (9, 10). Probiotics exert their health-promoting effects by changing the composition of the gut microbiome and increasing the number of *Bifidobacterium* and *Lactobacillus* species (11). Therefore, by protecting against colonization by pathogenic bacteria, probiotics enhance the gut barrier and reduce the risk of AD development (4).

Pre- and postnatal probiotic supplementation reduced the incidence of AD in infants and children (exposed *in utero* and up to 6 months after birth) (12). However, the benefits of probiotics in children and adolescents with established AD have not been studied. This study aimed to investigate the efficacy of a mixture of probiotics in improving the SCORAD in children and adolescents with AD. The secondary outcomes were to evaluate the use of topical and oral medicines (standard treatment), the role of probiotics on sensitization (serum IgE levels and skin prick test), inflammation (IFN-γ, IL-1β, IL-4, IL-6, IL-8, IL-17, and TNF-α), and tolerance (IL-10 and TGF-β).

## Materials and Methods

### Study Design

This parallel, randomized, double-blind, placebo-controlled trial was conducted between August 2015 and August 2016 at the Clinics Hospital of Ribeirão Preto Medical School, University of São Paulo, Brazil.

Enrolled patients received probiotics or placebo once a day for 6 months, and they continued to be followed up for 12 months.

### Patient Population

Patients aged between 6 months and 19 years with mild (SCORAD < 25), moderate (SCORAD 25-50), or severe AD (SCORAD ≥ 50), according to the criteria proposed by Hanifin & Rajka (13), were eligible for the study. Patients were selected among those who attended the Pediatric Allergy Outpatient Clinics at our hospital. Patients were enrolled if they experienced at least one documented AD flare treated with topical corticosteroids within the preceding 6 months. Exclusion criteria were other skin diseases that could interfere with the study; use of oral corticosteroids or immunosuppressants within 30 days before enrollment; and previous use of human monoclonal antibodies. Patients withdrew from the study if they experienced any severe adverse event during the study.

### Sample Size

The sample size was determined based on the comparison of the mean SCORAD between the probiotic and placebo groups at the end of the follow-up. By establishing a variance of 10.2 points (14), a clinically relevant difference of 8.7 points (15), a significance level of 0.05, and statistical power of 0.90, 60 patients were required to conduct the study.

### Randomization and Blindness

Patients were clinically assessed for AD severity (based on the baseline SCORAD) and stratified according to severity (mild, moderate, or severe), and randomly allocated to treatment or placebo by block randomization (blocks of random size 4) using a previously generated randomization list made in www.sealedenvelope.com by a statistician not involved with patient recruitment. The senior manager of the company who provided the probiotics and placebo (Farmoquímica S/A, Rio de Janeiro, Brazil) held the secret code for each group (A and B) until the end of the trial. Patients, caregivers, and investigators were blinded to treatment allocation throughout the study.

Enrollment, clinical assessments, laboratory analysis, and SPT were performed by the same researcher. The trial protocol was not modified after it was initiated.

### Interventions

A group of patients received a daily dose (once a day) of 1 g (sachet) of a mixture of four probiotic strains (Probiatop®): *Lactobacillus rhamnosus* HN001-10^9^ Colony Forming Units (CFU); *Lactobacillus acidophilus* NCFM-10^9^ CFU; *Lactobacillus paracasei* Lcp-37 - 10^9^ CFU; and *Bifidobacterium lactis* HN019-10^9^ CFU. The other group of patients started on 1 gram (once a day) of placebo (maltodextrin), as a sachet, to ensure similarity to the probiotics. Both sachets were identical, except for their identification as group A or B, to guarantee that the participants and the investigator were blind to their contents. The patients were instructed to dilute one sachet in 100 mL of water. The treatment lasted for 6 months. An external committee composed of three physicians who were not involved in the trial monitored the study.

### Clinical Assessment

All patients underwent clinical assessment at baseline, after 3 and 6 months of treatment (T3 and T6), and 3 and 6 months after treatment has been stopped (T9 and T12). Clinical assessments included a medical interview, physical examination, and SCORAD (primary outcome).

### Standard Treatment Assessment

The number of different oral and topical products (medicines) regularly used by each patient was recorded at each time point, but not the dosages.

### Cytokines Assessment

Blood samples were drawn at three different moments: at baseline, after 6 months of treatment (T6), and at the end of the follow-up (T12). SPT was carried out at baseline and the end of the follow-up (T12).

For cytokines analysis, peripheral blood was collected and centrifuged at 2,500 rpm for 10 min at 4°C. The serum samples were stored and frozen at −80°C until all analysis. Briefly, wells in a 96-well plate were covered and incubated with purified antibodies anti-IL-17 (R&D System, Minneapolis, MN, EUA), anti-IFN-γ, anti-IL-1β, anti-IL-4, anti-IL-6, anti-IL-8, anti-IL-10, anti-TNF-α, or anti-TGF-β (BD Bioscience, San Diego, CA, EUA), and enzyme-linked immune sorbent assays (ELISA) were carried out, according to the manufacturer's instructions.

### Sensitization Assessment

Serum total IgE levels were measured by a fluoroenzyme immunoassay (Phadia ImmunoCap System, Uppsala, Sweden), according to the manufacturer's instructions. The results were expressed in kU/L and were considered elevated when above 100 kU/L.

SPT was performed in all patients by using a panel of standardized extracts (Greer®): mites (*Dermatophagoides pteronyssinus, Dermatophagoides farinae*, and *Blomia tropicalis*), cockroaches (*Blatella germanica* and *Periplaneta americana*), pet's dander (cat and dog), and some food allergens (milk, egg, soybean, wheat, peanuts, seafood, and fish). The test was considered positive when a circle > 3 mm of diameter was visible 20 min after application.

### Ethical Considerations

This study was registered in the ClinicalTrials.gov (NCT 02519556) and was approved by the Ethics Committee of Clinics Hospital of Ribeirão Preto Medical School (protocol number 2367/2015). The trial was conducted following the Declaration of Helsinki and the International Conference on Harmonization Good Clinical Practice guidelines. Parents or legal guardians signed a written informed consent for their child's participation, whereas adolescents also signed an assent term.

### Statistical Analysis

SCORAD and serum IgE were analyzed as percent changes from baseline: the values observed at times 3, 6, 9, and 12 months were divided by the value observed at T0 and then multiplied by 100. The data were analyzed by adjusting simple and multiple linear mixed models; repeated measurements for the same individual were accommodated by a random effects model. The multiple models were adjusted for: age, sex, delivery type, and medications (moisturizers, antihistamines, topical corticosteroids, topical immunosuppressants, and antileukotrienes). To compare the frequencies of use of different topical and oral medicines for AD, Mann-Whitney tests were used. To assess the association of the intervention groups with the cytokines and SPT results, log-binomial regression models were proposed, as well as crude and adjusted relative risks (RR) and their 95% confidence intervals (CI) for the categories: improvement or worsening (in cytokine levels or in SPT result).

The analysis was per protocol. All the statistical analyses were performed with the software SAS 9.4.

## Results

### Baseline Description of Patients

**Figure 1:**
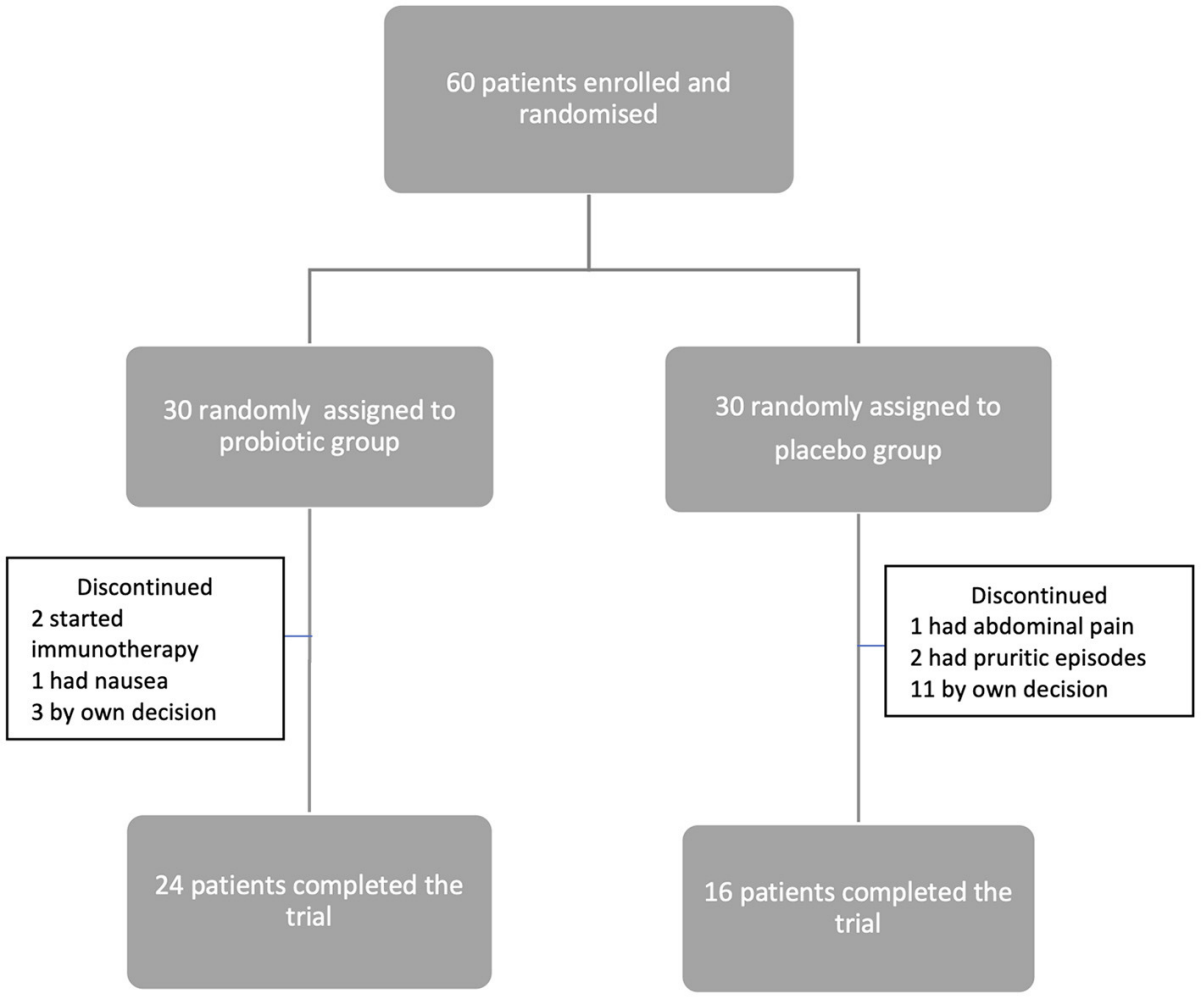
Flowchart of patient inclusion, allocation, and follow-up.

We followed the CONSORT recommendations for reporting randomized clinical trials. Sixty patients were enrolled in the study and randomized, and 40 completed the trial. Of those, 24 patients received probiotics and 16 patients received placebo (Figure 1). Recruitment was stopped when the required sample size was reached.

Table 1 shows the baseline demographic characteristics of the 40 patients who completed the study.

**Table 1:** Demographic characteristics of patients, per random group assignment.

	**Placebo**	**Probiotics**
	***n* = 16**	***n* = 24**
Sex		
Female	8	16
Male	8	8
Age distribution		
2–6 years	4	7
6–12 years	9	10
Adolescents	3	7
Delivery type		
Normal	5	11
Cesarean section	11	13
SCORAD, mean (range)		
Mild (<25)	13.86 (5.59–23.97)	15.64 (5.68–22.31)
Moderate (25–50)	36.83 (25.44–49.86)	33.49 (29.27–41.66)
Severe (≥50)	65.38 (50.36–80.42)	65.51 (54.37–85.53)
Total IgE, geometric mean UI/mL (range)	2130 (28.4–8660)	2413.01 (6.6–16000)

### Clinical Assessment

**Figure 2:**
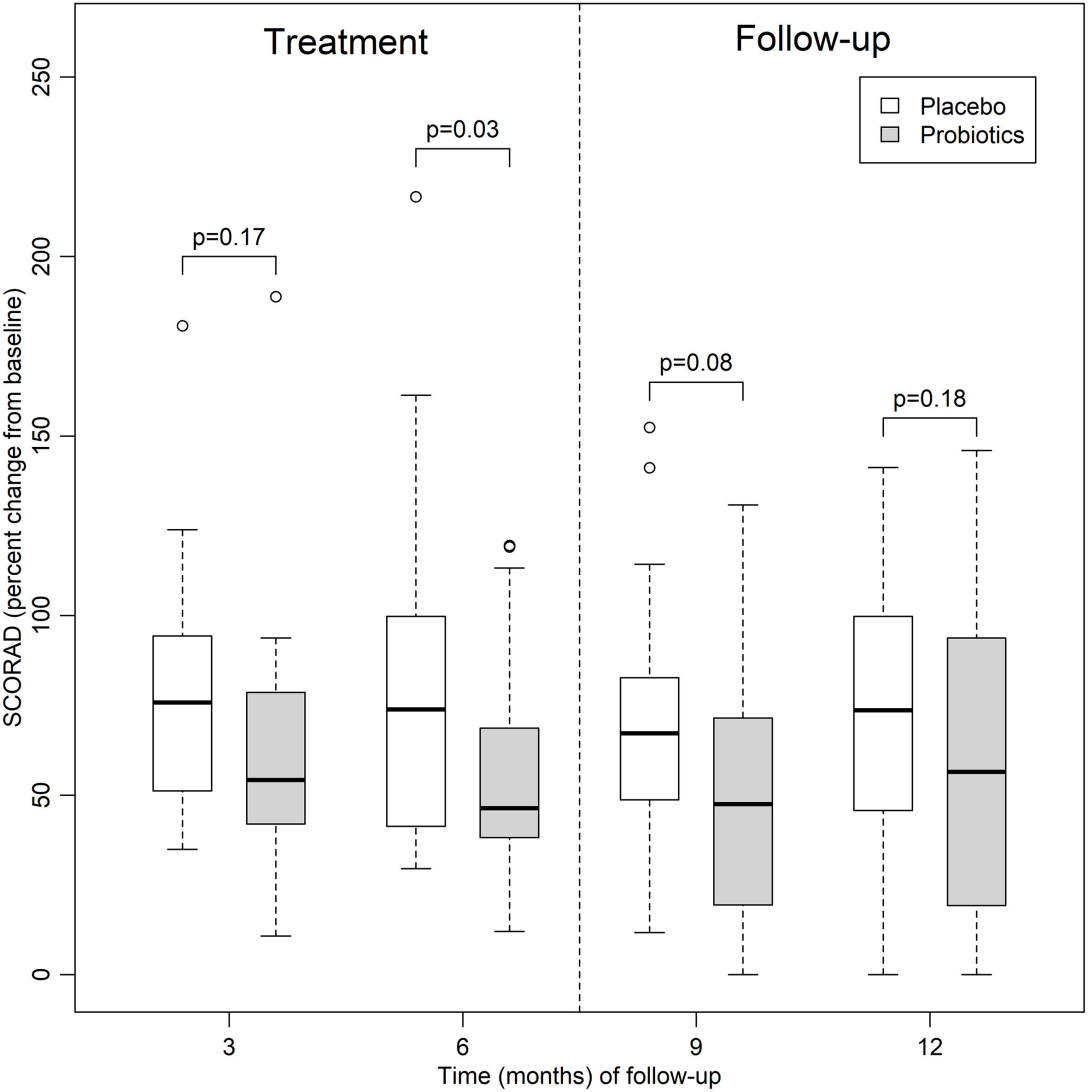
Relative percent changes in SCORAD (from baseline) in the probiotics and placebo groups up to 12 months of follow-up.

After treatment for 6 months (T6), patients in the probiotics group experienced a significant reduction in SCORAD as compared to patients in the placebo group (mean difference 27.69 percentage points; 95%CI, 2.44–52.94; Figure 2), This beneficial effect persisted after adjustment for the covariates (mean difference 32.33 percentage points; 95%CI, 5.52–59.13).

SCORAD decreased significantly from baseline in the probiotic group after 6 months of treatment and this reduction persisted for at least three more months (T9), after adjustment for the covariates (mean difference 14.24 percentage points; 95%CI, 0.78–27.70). However, this difference disappeared between T9 and the end of the study.

### Standard Treatment

**Figure 3:**
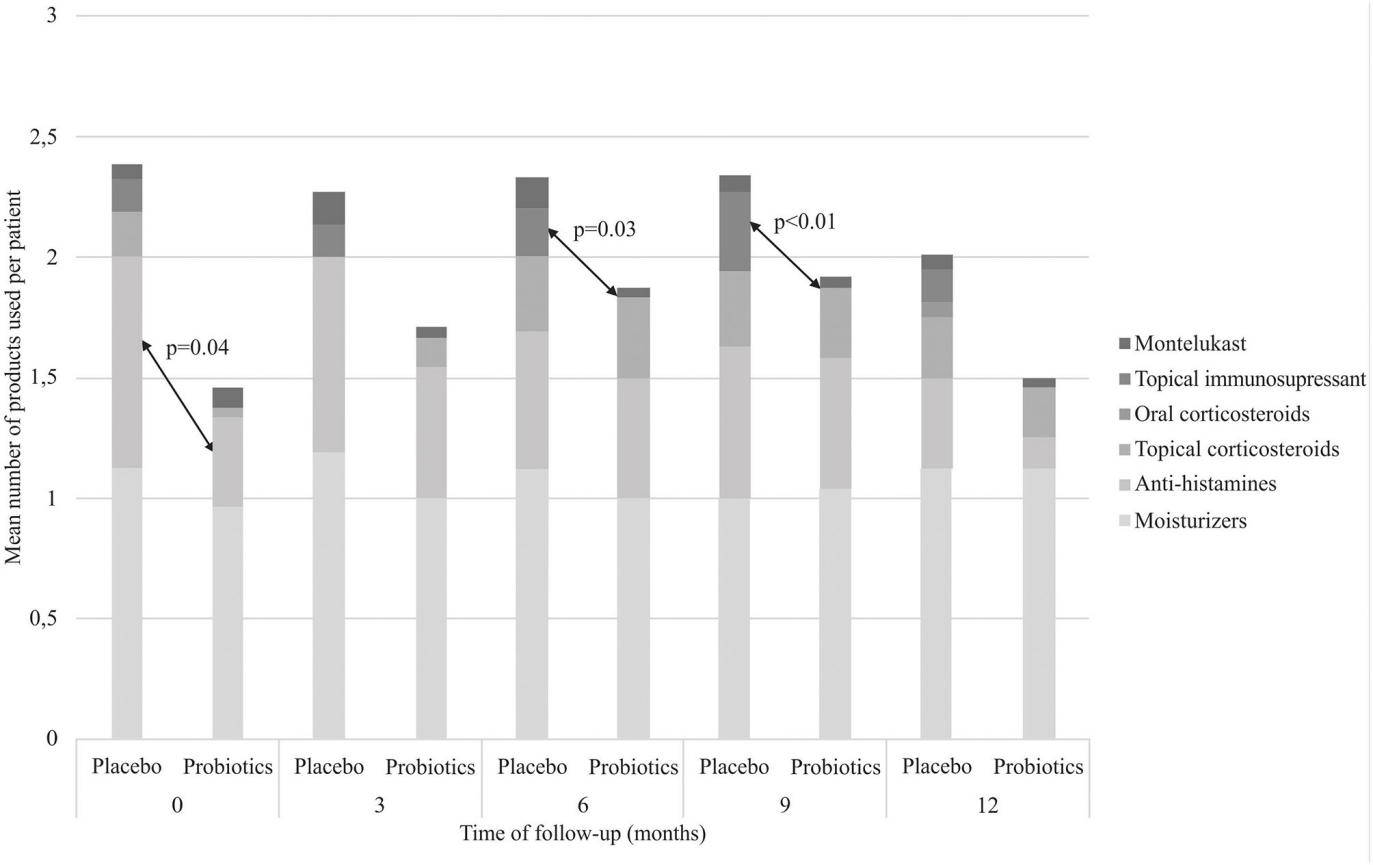
Mean number of medicines used by patients at each time point of follow-up.

Patients in the probiotics group used antihistamines less frequently at baseline and required topical immunosuppressant less frequently at 6 and 9 months (Figure 3, *p* < 0.05).

### Sensitization Assessment

**Figure 4:**
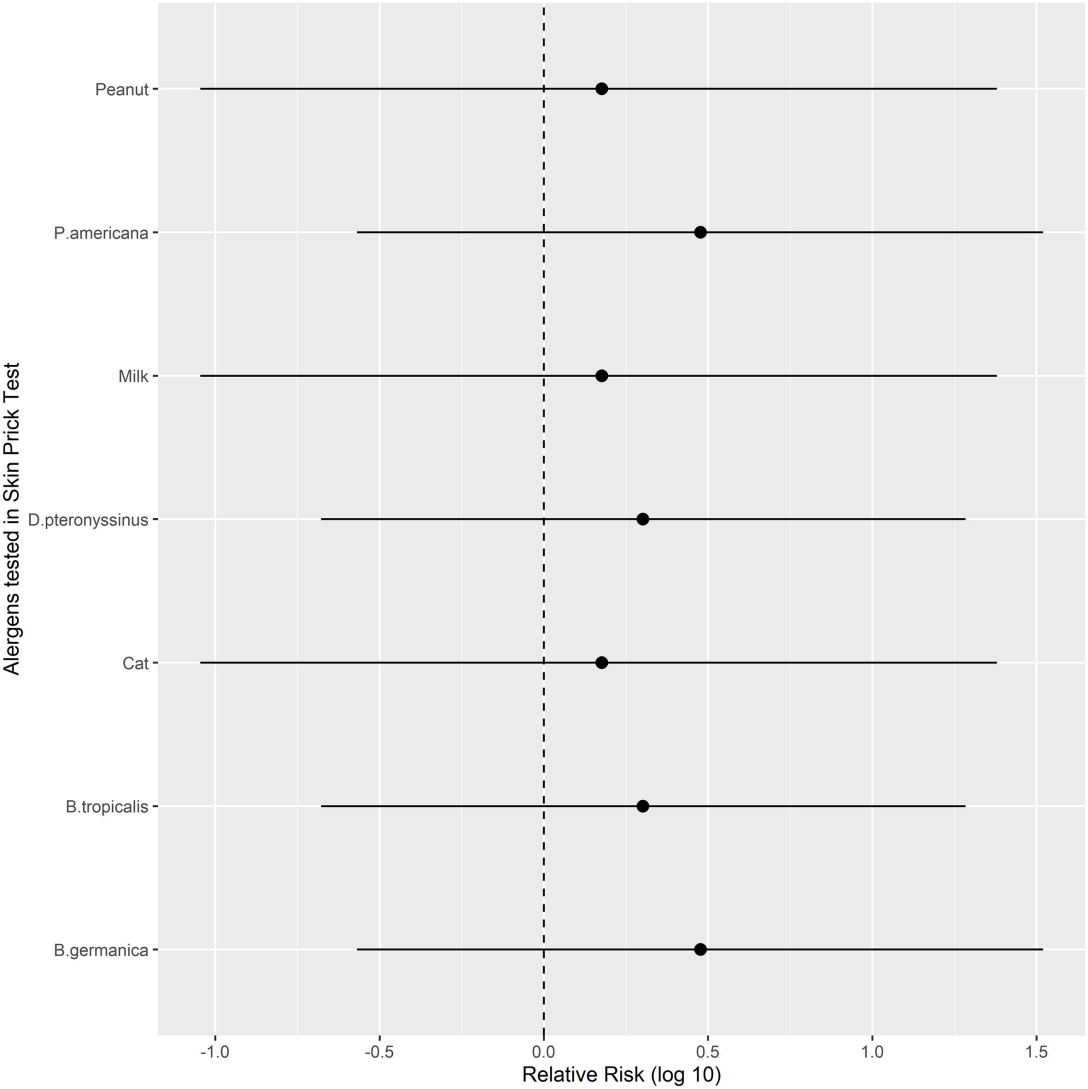
Relative risks (with 95% confidence intervals, log-transformed) of improvement in SPT results in the Probiotics group vs. Placebo for different antigens at the end of follow-up (T12). Bg, *Blatella germanica*; Bt, *Blomia tropicalis*; Dpt, *Dermatophagoides pteronyssinus*; Pa, *Periplaneta americana*.

Regarding the serum IgE levels (Table 2) and SPT results (Figure 4), the probiotics and placebo groups did not differ significantly.

**Table 2:** Serum IgE: comparison between Placebo (A) and Probiotics (B) groups.

		**Simple model** **95% CI**				**Adjusted model** **95% CI**		
**Comparisons**	**Mean difference**	**p**	**LL**	**UL**	**Mean difference**	**p**	**LL**	**UL**
A6–B6	0.04	0.93	−0.82	0.90	−0.03	0.95	−0.88	0.82
A12–B12	0.48	0.27	−0.38	1.33	0.41	0.33	−0.43	1.26

*A6, Placebo group after treatment for 6 months; B6, Probiotics group after treatment for 6 months; A12, Placebo group at the end of the study; B12, Probiotic group at the end of the study; LL, Lower limit; UL, Upper limit*.

### Cytokines Assessment

**Figure 5:**
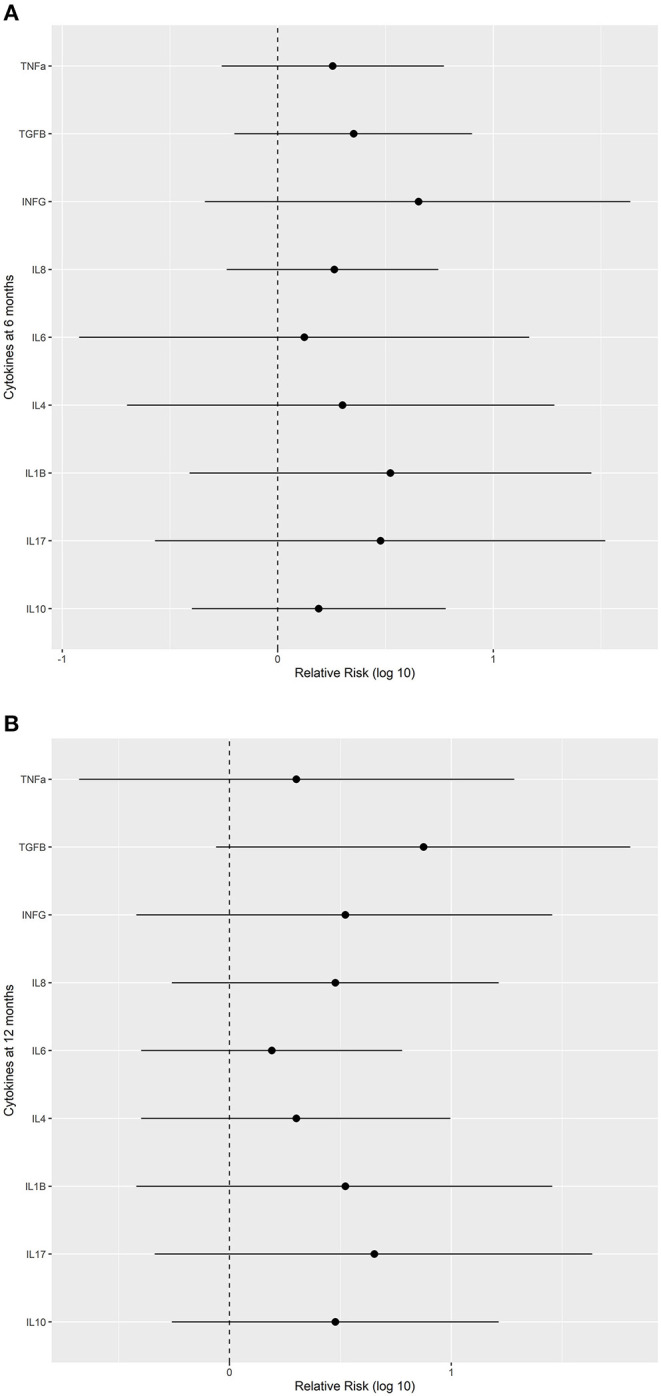
Relative risks (with 95% confidence intervals) of improvement in levels of selected cytokines in the Probiotics group vs. Placebo. **(A)** after treatment for 6 months (when treatment was discontinued) and **(B)** at the end of the study. IFN, interferon; IL, interleukin; TGF, transforming growth factor; TNF, tumor necrosis factor.

Regarding tolerogenic and inflammatory cytokines, the relative risk was not significantly different between the groups (Figure 5).

### Adverse Events

The most commonly reported adverse events were nausea, abdominal pain, and worsening pruritic episodes (1 patient in probiotic group and 3 patients in placebo group), as shown in Figure 1. No severe adverse events were observed.

## Discussion

In this randomized, double-blind, placebo-controlled study, we assessed the clinical and laboratory effects of a mixture of probiotics containing *Lactobacillus rhamnosus, Lactobacillus acidophilus, Lactobacillus paracasei*, and *Bifidobacterium lactis* in children and adolescents with AD. We demonstrated that the regular use of probiotics for 6 months promoted a statistically significant reduction in SCORAD in those patients even after adjustment for covariates. This improvement was statistically significant after the third month of treatment. We also found that the probiotics group required topical immunosuppressant less frequently at 6 and 9 months. However, the groups did not differ regarding SPT, serum IgE levels, and cytokine profile. To our knowledge, this is the first Brazilian randomized clinical trial that has demonstrated significant clinical benefits of probiotics in pediatric patients with AD.

Our sample of patients consisted predominantly of children (75% of the patients), as expected (16). The prevalence of allergic diseases has increased in the last decades in both high- and low-income countries (17). In the past, AD was traditionally considered a childhood disease (18), with a prevalence of 10–20% (12); however, recent studies have shown that AD may persist for the whole life (18). Because AD has high morbidity, the interest in this disease has increased (19). This point reinforces the need for an accessible, safe, and effective treatment for AD that can minimize or override the long-term effects of skin inflammation.

We found that almost half of our patients had mild disease (47.5%), but a significant percentage of the patients had moderate disease (37.5%). This could be explained by the fact that this study was conducted in a tertiary-care hospital. On the other hand, the low percentage of patients with severe AD (15%) could be due to the exclusion criteria adopted herein (treatments that interfere with systemic inflammation, such as the use of systemic immunosuppressants and monoclonal antibodies), which are very common among severe patients. Most AD patients are diagnosed with mild disease during childhood (2). In a 4-year prospective study, Kim et al. (20) found mild severity in 70% of the children with AD.

In the present study, a higher number of children were delivered by cesarean section (60%), which may have influenced their predisposition to AD; however, we have not evaluated the patient's microbiota. The high AD prevalence in developing countries over the four last decades has been attributed to excess hygiene, which reduces the exposure of the immune system to microbes (21, 22). Factors like early-life antimicrobial exposure, formula feeding, and maternal use of antimicrobials during pregnancy affect the microbiota composition, thus contributing to allergic disease development (5). The amount and type of commensal microbes in the human gut during the neonatal period are shaped by early life exposures (23). Even the mode of birth can cause alterations in the gut microbiota that lead to altered immunologic responses (23). Jakobsson et al. (24) demonstrated that children born by cesarean section had poorer diversity of *Bacteroides*. Antisepsis related to the surgical procedure and lack of exposure to the maternal vaginal microbiota may cause this low colonization (23).

We demonstrated that the patients on regular use of a mixture of probiotics (*Lactobacillus* and *Bifidobacterium*) for 6 months presented a statistically significant reduction of SCORAD and used less topical immunosuppressants as compared to the placebo group, especially from the third month of treatment on. This reduction persisted up to 3 months after the treatment was discontinued, suggesting that the clinical benefit is related to treatment duration and that the probiotics maintain a short-term beneficial effect after the treatment is discontinued. There is growing evidence that the relationship between gut microbiota and immune response can play a role in allergic diseases (22). Recently, several clinical trials have suggested that the administration of specific probiotics in early life could reduce the risk of AD (12). In general, using probiotics before and after birth is beneficial, and *Lactobacillus rhamnosus GG* and *Bifidobacterium* seem to be the most efficient strains (25). Han et al. (26) performed a 12-week randomized, double-blind, placebo-controlled study to evaluate the role of *Lactobacillus plantarum* CJLP133 in children with AD aged between 1 and 12 years. The authors showed that SCORAD improved and that IFN-γ, IL-4, and the number of eosinophils decreased. In another study, Wang et al. (27) evaluated how *Lactobacillus paracasei, Lactobacillus fermentum*, and the association of both strains affected AD patients. After treatment for 4 months, the authors observed a reduction of SCORAD in all groups that received probiotics as compared to the placebo group. Huang et al. (28) carried out a meta-analysis that included thirteen randomized clinical trials (*n* = 1,070 patients), to show that probiotics may decrease SCORAD values in children with AD. Navarro-Lopez et al. (29) published a double-blind, placebo-controlled clinical trial conducted with children and adolescents with moderate AD aged between 4 and 17 years. The patients were supplemented daily with a mixture of probiotics composed of *Bifidobacterium lactis* CECT 8145, *Bifidobacterium longum* CECT 7347, and *Lactobacillus casei* CECT 9104 for 12 weeks. SCORAD and the use of topical corticosteroids decreased. Suzuki et al. (30) found that daily ingestion of yogurt containing *Lactococcus lactis* 11/19-B1 for8 weeks promoted a significant reduction of SCORAD in children with AD.

In the present study, we found that the probiotics group used less topical immunosuppressants compared to the placebo group. Because topical corticosteroids and calcineurin inhibitors are recommended as the standard treatment of AD in several guidelines, evaluation of efficacy of probiotics and any other complementary therapy can be performed in AD patients as an outcome. Moroi et al. (31) performed a randomized, double-blind, placebo-controlled study with AD adults who were treated with conventional topical corticosteroid and tacrolimus. The consumption of topical therapeutics in the placebo group was 1.9-times greater in total amount compared with the corresponding value in the *Lactobacillus paracasei* K71 diet group during the intervention period (12 weeks), although there was no significant difference.

Despite the reduction of SCORAD in all of our patients of the probiotics group, some of them presented more significant clinical improvement than others. Avershina et al. (21) analyzed fecal samples obtained from children aged 2 years whose mothers had been submitted to supplementation with probiotics during pregnancy. The authors showed that the gut microbiota from children with AD and good clinical response was comparable with children without AD. In contrast, the group with AD and poor clinical response had a different microbiota. Therefore, the clinical effects of probiotics may depend on the host's microbiota and could explain the better response verified in some of our patients compared to others. Future studies analyzing fecal microbiota will help to elucidate this hypothesis.

Two main biological pathways are involved in AD pathogenesis: epidermal dysfunction and imbalance in innate/adaptive immune response (22). T helper cells play an important role in inflammation. Regulatory T cells (Treg) play a central role in immune tolerance maintenance. Reduced Treg number in early life is a risk factor for later AD development (19). In the acute phase, Th2 cell-mediated immune response triggers the inflammatory process. On the other hand, Th1 cell-mediated immune response predominates in the chronic phase. Some studies have suggested that Th17 cells are also involved in AD pathogenesis (16). There is evidence that probiotics can modify the course of the immune response. Cao et al. (32) demonstrated that probiotics downregulated the function of Th_2_ cytokines in allergic rats. Previous studies reported that some strains like *Lactobacillus rhamnosus* GG and *Lactobacillus paracasei* KW3110, improved AD through regulation of Th1/Th2 balance and anti-inflammatory response (30). Holowacz et al. (33) showed that a mixture of probiotics promoted a significant reduction of chronic skin inflammation in rats, confirmed by the lower levels of pro-inflammatory cytokines (IL-1β, IL-6, TNF-α, IL-17, and IL-22) and higher levels of tolerogenic cytokines, such as IL-10. A recent study in mice demonstrated that *Lactobacillus lactis* 11/19-B1 intake suppressed Th1, Th2, and Th17 cells in Peyer's patches and cervical lymph nodes, instead of Treg cells (30).

However, the effects of probiotics on T cells subsets and cytokines in children with AD are conflicting. Some studies have reported a tendency toward the Th1 profile resulting in increased interferon-gamma (IFNγ) production, whereas other studies have not shown any effect on Th1/Th2 balance or Treg (19). Yeşilova et al. (34) performed a double-blind, randomized, placebo-controlled clinical trial to investigate how a combination of probiotics (*Bifidobacterium bifidum, Lactobacillus acidophilus, Lactobacillus casei*, and *Lactobacillus salivarius*) affected SCORAD and cytokine profile in children with AD aged between 1 and 13 years old. The treatment effectively reduced SCORAD and serum levels of IL-5, IL-6, IFN-γ, and IgE, while IL-2, IL-4, TNF-α, and IL-10 remained unaltered. On the other hand, Ludwig et al. (35) found that *Lactobacillus rhamnosus* GG soluble mediators (LSM) did not modify the maturation stage or the number of dendritic cells in healthy donors; nevertheless, these cultivated cells induced IFN-γ and IL-2 production in CD4^+^ and CD25^+^ cells. In the present study, we did not find any interference of probiotics on inflammatory and tolerogenic cytokines. Although we did not carry out cell culture, our assessment included a panel of cytokines that represents all the immune responses pathways (Th1, Th2, Th17, and Treg). We can point some possible explanations. Yeşilova et al. (34) used a higher dose of probiotics (2 g) and the cytokines were measured in the plasma, whereas we analyzed cytokines in the serum. Besides that, studies that analyzed cytokines in children with AD who underwent treatment with probiotics are scarce, which prevents us from drawing more conclusions about other possible reasons for these results.

We did not find any effect of probiotics on serum IgE levels or SPT results, either. We did not find any other studies evaluating SPT in AD patients treated with probiotics.

In our study, probiotics were well tolerated and no severe adverse events were observed. The most common adverse events were nausea, abdominal pain, and worsened pruritic episodes, especially in the placebo group. One patient of the probiotics group presented nausea. Probiotics, especially Lactobacilli and Bifidobacteria (36), are safe (4). The most commonly described adverse events are diarrhea, vomiting, and increased flatulence (4). Although invasive infections have been observed in immunocompromised adults, they are rare in breastfed infants (36). However, according to a recent systematic review and a meta-analysis performed by Zhao et al. (37), the safety profile of probiotic treatment was not adequately studied.

The main strength of this study is that all patients were evaluated by the same investigator throughout the study, providing early recognition of possible complications and allowing for more personalized care. This may have improved the accuracy of SCORAD assessments throughout the study. Moreover, few clinical trials explored the effects of *Bifidobacteria* in combination with *Lactobacillus* in children with AD.

We can point some limitations of the study. Treatment duration may not have been optimal. Our daily dose of probiotics (1 g) was lower than that of the previous study by Yeşilova et al. (34) (2 g). The number of patients who completed the study was relatively small; however, it was sufficient to demonstrate a significant clinical improvement of disease with probiotics as compared to placebo, especially from the third month of treatment on.

Future studies about the role of probiotics on the immune response and intestinal microbiota will help to understand AD pathogenesis and to elucidate the controversies, thereby contributing to alternative therapies.

Some evidence indicate the possibility of influencing the composition of breast milk by taking probiotics by pregnant and lactating women. A relationship was observed between maternal supplementation with multi-component probiotic preparations and IL6 mean values in colostrum and between IL10 and TGF-β1 mean values in mature breast milk (7).

Studies on the effect of probiotic supplementation by pregnant and lactating women showed a relationship between the amount of Lactobacilli and Bifidobacteria in the colostrum and mature milk of mothers receiving probiotic preparation with vaginal delivery compared to mothers receiving placebo. Also, the study conducted by Abrahamsson et al. (38) has shown the ability to transfer the Lactobacillus bacteria in breast milk after oral supplementation of women in the final stages of pregnancy. The microbiota of breast milk affects the microbiota of the child's digestive tract, and thus is an important factor in maintaining body homeostasis, preventing many diseases in both the short and long term (7). The future role of probiotics in the infant health warrants further investigation.

In conclusion, our study demonstrated that children and adolescents with AD treated with a combination of probiotics (*Lactobacillus rhamnosus* HN001-10^9^ CFU; *Lactobacillus acidophilus* NCFM-10^9^ CFU; *Lactobacillus paracasei* Lcp-37-10^9^ CFU; and *Bifidobacterium lactis* HN019-10^9^ CFU) for 6 months presented a statistically significant reduction of SCORAD and used less topical immunosuppressants as compared to the placebo group. This reduction persisted for 3 months after the treatment has been discontinued. Our results warrant further studies to investigate optimal duration and dose as well as the long-term efficacy and possibility of sustained effects of probiotics in patients with AD.

## Data Availability Statement

The raw data supporting the conclusions of this article will be made available by the authors, without undue reservation.

## Ethics Statement

The studies involving human participants were reviewed and approved by Ethics Committee of Clinics Hospital of Ribeirão Preto Medical School. Written informed consent to participate in this study was provided by the participants' legal guardian/next of kin.

## Author Contributions

PA and PR-J: study design and implementation. PA, JM, and PR-J: clinical assessment. PA, VC, LS, and LR: lab work. DA and FC: statistical analysis. PA, DA, FC, and PR-J: data analysis and interpretation. All authors substantially approved the final manuscript.

## Funding

The Probiatop® and placebo samples as well as cytokines kits were provided by Farmoquímica S/A. Funding sources had no influence in study design; in the analysis and interpretation of data; in the writing of the report; and in the decision to submit the manuscript for publication.

## Conflict of Interest

The authors declare that the research was conducted in the absence of any commercial or financial relationships that could be construed as a potential conflict of interest.

## Publisher's Note

All claims expressed in this article are solely those of the authors and do not necessarily represent those of their affiliated organizations, or those of the publisher, the editors and the reviewers. Any product that may be evaluated in this article, or claim that may be made by its manufacturer, is not guaranteed or endorsed by the publisher.
